# The Cost-Effective Preparation of Green Fluorescent Carbon Dots for Bioimaging and Enhanced Intracellular Drug Delivery

**DOI:** 10.1186/s11671-020-3288-0

**Published:** 2020-03-04

**Authors:** Yuqing Sun, Shaohui Zheng, Long Liu, Ying Kong, Aiwei Zhang, Kai Xu, Cuiping Han

**Affiliations:** 1 School of Medical Imaging, Xuzhou Medical University, Xuzhou, Jiangsu 221004 People’s Republic of China; 2 Department of Radiology, Affiliated Hospital of Xuzhou Medical University, Xuzhou, Jiangsu 221000 People’s Republic of China

**Keywords:** Green fluorescent carbon dots, Bioimaging, DOX-CDs, Targeted delivery

## Abstract

Doxorubicin entrapped carbon dots (DOX-CDs) were prepared for bioimaging and enhanced intracellular drug delivery. The CDs were synthesized via the hydrothermal method using citrate and urea under 200 °C for 1 h. Then, DOX was successfully conjugated on the CDs via physicochemical interactions. The DOX-CDs exhibited good crystal structure, remarkable aqueous stability, excellent photoluminescence property, and a high quantum yield of 93%. The fluorescent images revealed that the DOX-CDs could be readily taken up by the cancer cells for cell labeling. Furthermore, endo-lysosomal pH-assisted DOX release behavior was observed from DOX-CDs, and the cytotoxicity of DOX-CDs was confirmed by the MTS assay against H0-8910 ovarian cancer cells. In addition, the CDs indicated bright fluorescent signal in the animal imaging test and demonstrated low toxicity after administration for 7 and 21 days. Therefore, the prepared CDs could be a promising imaging probe for biomedical imaging and intracellular drug delivery.

## Introduction

Doxorubicin (DOX) is an anthracycline chemotherapeutic drug widely used in the treatment of several cancers including breast, lung, gastric, ovarian, thyroid, multiple myeloma, sarcoma, and pediatric cancers. The mechanism of anticancer action for DOX is considered as interfering DNA synthesis and repair process. Therefore, DOX needs to be transported across the cell membrane and to cell nucleus to disturb DNA synthesis during cancer treatment. However, free DOX could not easily approach cell nucleus and would induce severe in vivo cardiotoxicity, which hindered its application as cancer therapy [1, 2].

Recently, multifunctional nanocarriers such as liposomes, micelles, nanoemulsions, polymeric nanoparticles, and other nanoparticles have attracted tremendous attention for their significance on anticancer drug delivery [3]. Among them, as a new type of quantum dots family, carbon quantum dot (CD) has drawn enormous interest worldwide since it was discovered in 2004 [4]. In particular, fluorescent CDs have become the preference in cell imaging^2^, photocatalysis, drug delivery, pollutants and heavy metal ions detection, and photoelectrical equipment due to their superior properties in quasi-zero weight and size (< 10 nm), high photo-stability, broad and continuous excitation spectra, tunable wavelength, satisfying biocompatibility, low toxicity, and excellent performance on fluorescence [5–14]. For example, Yang et al. have successfully coupled DOX to the CDs for enhanced anticancer treatment, implying that CDs have a great significance on nucleus-targeted drug delivery [1].

Previously, various techniques have been proposed to prepare CDs including numerous biomass materials, carbonization, passivation, and surface functionalization [14, 15]. In detail, it can be classified as two main methods. One is the “top-down” approach, arc discharge, laser ablation method, electrochemical etching, and oxidation method, which refers to breaking the large scale of carbon structure into carbon nanoparticles [16–20]. The other method is the “bottom-up” method, which synthesizes carbon dots from molecular precursors, mainly including hydrothermal approach, ultrasonic method, and wave-assisted synthesis [21–24]. Xu et al. obtained carbon dots through arc discharge, oxidation, extraction, and gel electrophoresis. Ming et al. acquired carbon dots by electrolysis. Yang et al. optimized the original hydrothermal method to synthesize carbon dots with different fluorescence [21]. However, these methods are limited owing to their complicated synthesis process, time-consuming procedure, strict fabrication requirements, and expensive raw materials [25]. Moreover, the use of organic solvents as passivators for the reaction can increase the toxicity of CDs [26]. Besides, most of the reported CDs emitted blue fluorescence under UV light excitation which seriously hindered their potential in the field of biomedical imaging attributed to the strong tissue autofluorescence interfering.

Herein, we synthesized green fluorescent CDs via a green and efficient one-step controlled thermal pyrolysis of sodium citrate dihydrate and urea. DOX was non-covalently conjugated on the surface of prepared CDs for drug delivery by means of hydrophobic interaction and electrostatic interaction, as well as *π*-*π* stacking interaction [27–29]. The morphology and structure of CDs were investigated by transmission electron microscopy (TEM) and X-ray diffraction analysis. The optical properties were assessed by UV-vis spectrometer and photoluminescence (PL) emission spectra. The sustained drug release was performed by the dialysis method. The cellular uptake and the intracellular distribution of DOX-CDs were investigated by a fluorescent microcopy. The anticancer effect of DOX-CDs was assessed by standard MTS assay. The in vivo imaging of CDs was carried out on Balb/c nude mice. Finally, the long-term toxicity of CDs was investigated by the histological analysis. Therefore, the prepared CDs would be a potential agent for in vivo imaging and targeted drug delivery.

## Materials and Methods

### Materials

Sodium citrate dihydrate, urea, l-arginine, ethylenediamine acetone, quinine sulphate, phosphate-buffered saline (PBS), acetic acid, disodium hydrogen phosphate, and paraformaldehyde were obtained from Sinopharm Chemical Reagent Co. Ltd (Shanghai, China). MTS Cell Proliferation Colorimetric Assay Kit (MTS), Dulbecco’s Modified Eagle Medium (DMEM/high glucose), penicillin-streptomycin solution, and trypsin-EDTA solution were purchased from Beyotime Biotechnology Co. Ltd (Shanghai, China). Fetal bovine serum (FBS) was obtained from Tianhang Biotechnology Co. Ltd (Hangzhou, China). The HO-8910 ovarian cancer cells and EA.hy926 human umbilical vein endothelial cells were obtained from Shanghai Institute of Nutrition and Health, Chinese Academy of Sciences (Shanghai, China). Doxorubicin hydrochloride (DOX) was purchased from Sigma-Aldrich (Shanghai, China). The dialysis bags (MWCO = 1000 Da) were purchased from SpectrumLabs (Los Angeles, CA, USA).

### Synthesis of CDs and DOX-CDs

Briefly, sodium citrate dehydrate (0.2 mmoL) and urea (5 mmoL)were firstly dissolved in 1 mL DI water. Then, the mixture was transferred into a glass vessel and carbonized at 200 °C for 1 h. Thereafter, 1 mL DI water and acetone (v/v, 1/3) were added and centrifuged at 10000 rpm for 10 min for three times.

DOX was conjugated on the CDs by the noncovalent interaction. Briefly, DOX·HCl (0.5 mg) was added to 5 mL of CDs (5 mg/mL) and then stirred for 48 h in the dark. The resultant solution was dialyzed against DI water in a dialysis bag (MWCO = 1000 Da) for 24 h to obtain a DOX-CDs. Finally, the DOX-CDs were freeze-dried and stored under 4 °C.

### Characterization of CDs and DOX-CDs

The size morphology of CDs was characterized by transmission electron microscopy (TEM, FEI Tecnai G2 Spirit). The PL emission measurements were taken on a LS55 fluorescence spectrophotometer (PerkinElmer, Waltham, MA, USA). The quantum yield (*QY*) of CDs was determined by using quinine sulfate solution in H_2_SO_4_ as reference. The crystal structure was observed by a Bruker Tensor27 Fourier transform infrared spectrophotometer (Pike Corporation, Madison, Wisconsin). X-ray photoelectron spectroscopy (XPS) was performed using a ESCALAB250Xi spectrometer (Thermo, USA). Zeta potential was measured with a Zeta potentiometer (Malvern Panalytical, Malvern, UK).

### In Vitro Drug Release Study

The in vitro drug release of DOX from DOX-CDs was investigated by using a dialysis bag. Briefly, DOX-CDs were loaded into the dialysis bag and immersed in PBS (pH 7.4 and 5.0), respectively, then were placed in a shaking incubator (37 °C, 100 rpm). At the predetermined time, 0.5-ml samples were withdrawn and replaced with same volume of PBS. The released DOX was recorded by fluorescence intensity at 590 nm.

### In Vitro Cytotoxicity Test

The cell cytotoxicity of DOX-CDs was determined by MTS assay against HO-8910 ovarian cancer cells and EA.hy926 umbilical vein endothelial cells [30, 31]. Briefly, the cells were seeded in a 96-well plate at a concentration of 0.5 × 10^5^ cells/mL, maintained for 24 h to allow cell attachment. Then, DOX-CDs at various concentrations were added into each well. After 24 h incubation, the medium was aspirated, and 90 μL of the medium and 10 μL of MTS were added to each well. After 4 h, the absorbance at 490 nm was measured using a microplate reader (BioTek Epoch, Service Card). The cell viabilities were expressed as a percentage of survival cells and reported as the means of triplicate measurements.

### In Vitro Cell Imaging Study

The HO-8910 ovarian cancer cells were inoculated on 6-well plate and incubated at 37 °Cfor 24 h for cell attachment. Then, the cells were incubated with DOX-CDs to allow cellular uptake. After 4 h incubation, the medium was removed, and cells were washed thrice with cold PBS and fixed with 4% paraformaldehyde for 10 min. Finally, the morphology and fluorescence distribution of cells were visualized by a fluorescent microscope (Leica Microsystems, Wetzlar, Hessen, German).

### In Vivo Imaging

Animal experiments were approved by the Animal Care Committee of Xuzhou Medical University [32]. Balb/c nude mice were used to assess the potential of CDs in fluorescent imaging. Briefly, Balb/c nude mice were subcutaneously injected with CDs aqueous solution (50 μL, 6 mg/mL) at the injection site after intraperitoneal injection of 2% pentobarbital for anesthesia. Furthermore, the biodistribution of the CDs in the mice body was also investigated by injecting the CDs (5 mg/kg) via the tail vein. Different organs (heart, renal, spleen, liver, bladder) were collected for the fluorescence assessments at various time points. The animal fluorescence imaging was taken on a Tanon-5200Multi Gel imaging system, and the exposure time was 1.0 s for all fluorescence images.

### In Vivo Toxicity Study

Kunming mice (female, 7 weeks) were used to investigate in vivo long-term toxicity of CDs. Kunming mice were randomly divided into 2 groups: CDs and control group. The mice were injected with PBS and CDs via tail vein (6 mg/kg). Then, the main organs including heart, lung, kidney, liver, and spleen were collected after 7 and 21 days of injection. Thereafter, the organs were fixed with 4% paraformaldehyde, sliced, and stained with hematoxylin and eosin (H&E). Finally, the histological sections were observed under an optical microscope (Leica Microsystems, Wetzlar, Hessen, German).

## Results and Discussion

### Characterization of CDs and DOX-CDs

**Scheme 1:**
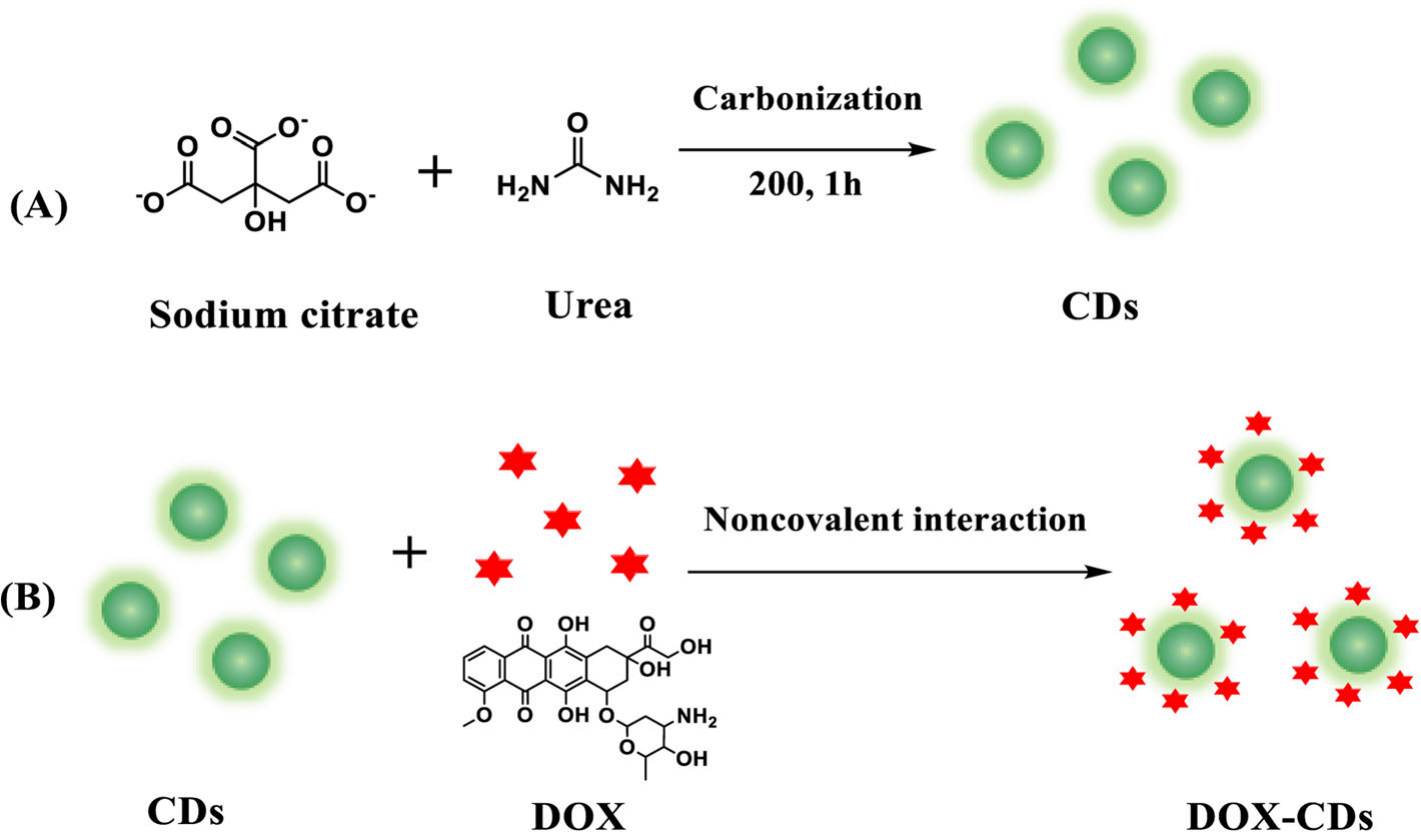
Schematic preparation of CDs (**a**) and DOX-CDs (**b**)

**Fig. 1:**
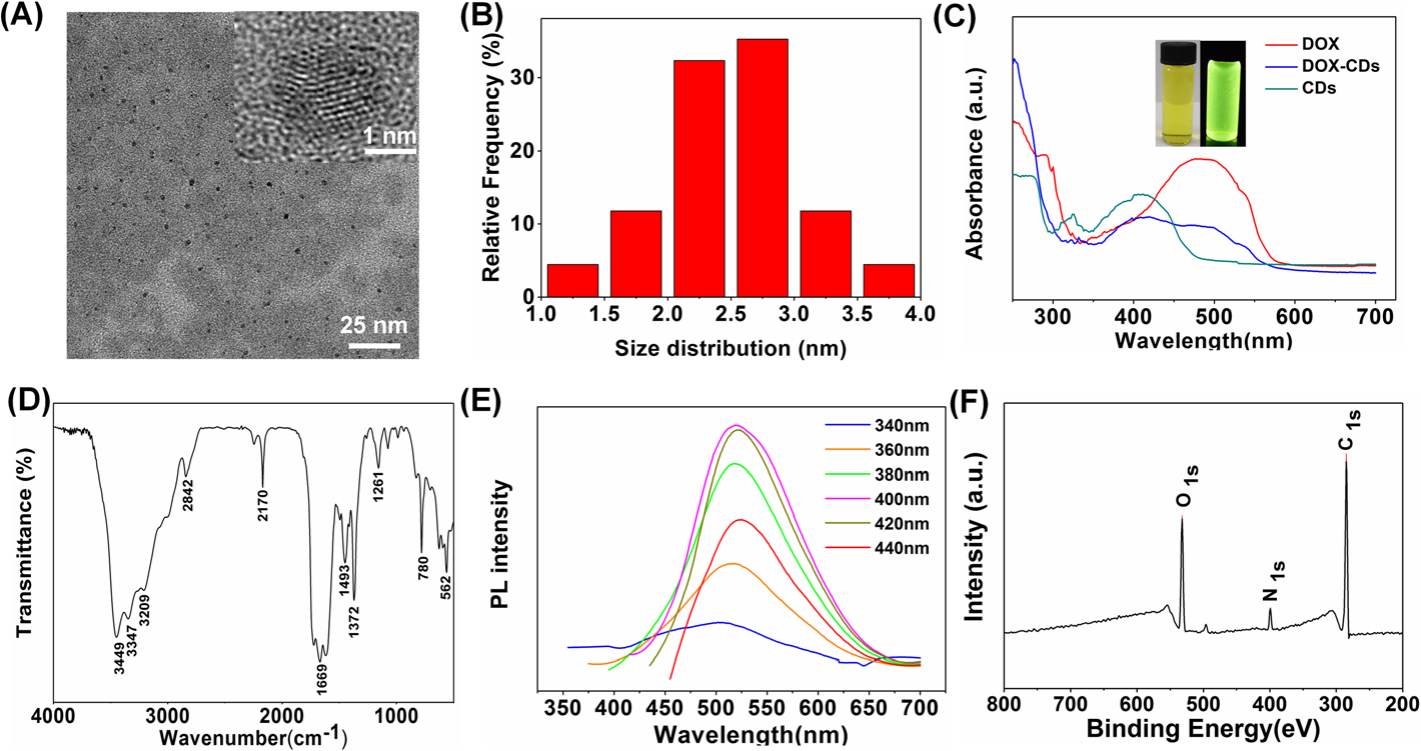
Characterizations of CDs. **a** TEM images of CDs (inset, high-resolution TEM images). **b** Size distribution of CDs. **c** UV-vis absorption of DOX, CDs, and DOX-CDs and inset pictures show the CDs under natural light and UV light. **d** FTIR spectra of CDs and **e** The PL emission of CDs with excitation wavelengths from 340 nm to 440 nm in 20 nm increments. **f** XPS spectrum of CDs

CDs were prepared through one-step strategy using sodium citrate dehydrate and urea (sodium citrate dehydrate/urea = 1/25) at 200 °C for 1 h. DOX was covalently conjugated on the surface of prepared CDs for drug delivery (Scheme 1). As shown in the TEM image (Fig. 1a), the CDs exhibited uniform spherical morphology with an average diameter of 2.75 nm and relatively narrow size distribution. In addition, the crystal structure of CDs could be observed by the high resolved TEM image (Fig. 1a inset), indicating well crystalline with noticeable lattice fringes.

The chemical structure of CDs was characterized by FTIR spectroscopy. As shown in Fig. 1d, the sharp peaks at 3499 cm^−1^ and 1729 cm^−1^ assigned to –OH and –COOH respectively, while those at 780 cm^−1^ and 1372 cm^−1^ could be attributed to N–H. It can be concluded that both carboxyl and amino groups exist on the surface of the carbon dots as functional groups and modify biological macromolecules with specific functions, which provides a possibility for further application research of the carbon dots.

Furthermore, the optical properties of CDs were investigated using UV-vis absorbance and PL spectroscopy. As shown in Fig. 1c, the CDs exhibited an absorbance peak at 410 nm, and DOX revealed an absorbance peak at 500 nm. Whereas, the DOX-CDs maintained the absorbance peaks of CDs and DOX at 410 nm and 500 nm, respectively, indicating the successful conjugation of DOX on CDs. Moreover, as shown in the inset figure of Fig. 1c, the DOX-CDs aqueous solution revealed light yellow and transparent under natural light and nut turned to bright green under UV excitation. Furthermore, fluorescence quantum yield was calculated to be 93% with quinine sulfate used as reference (*QY* = 54%). As CDs were excited at wavelengths from 340 to 440 nm, the PL peak showed nearly no shift, indicating the excitation-independent emission properties of CDs. The maximum excitation wavelength and PL peak of the CDs are 400 and 525 nm, respectively. The excitation-independent PL behavior may result from the uniform surface states of CDs [33].

In addition, the elemental composition of CDs was determined by XPS. As it is demonstrated in Fig. 1f, the XPS spectrum has determined that the CDs mainly constituted of carbon (*C*), nitrogen (*N*), and oxygen (*O*) and the corresponding atomic ratio of which was 78.39%, 7.52%, and 14.1%, separately. Three typical peaks of *C*_1S_, *N*_1S_, and *O*_1S_ can be observed at 284.8, 399.5, and 532.6 eV, respectively. To be specific, *C*_1S_ spectrum displayed 3 peaks at 284.8, 286.7, and 288.3 eV, representing the existence of C–C, C–N, C–O, or C=O bond, separately (Figure S1A). The high-resolution spectrum for *N*_1S_ revealed a peak at 399.5 eV, which were assigned to C–N. Moreover, the *O*_1s_ spectrum also confirmed the presence of C=O and C–O bond at 531.9 and 532.6 eV, respectively (Figure S1B).

The fluorescence intensity of CDs maintained wonderful stability both at 4 °C and room temperature (Figure S2A). The decrease of fluorescent intensity at 4 °C in 2 weeks for less than 10% can be neglected. Therefore, it is expected that the carbon dots possessed a long-term stability to be used as a biomedical imaging agent.

Figure S2B illustrated that PL intensities of CDs decreased in aqueous solutions of high (> 10) or low (< 3) pH. Nevertheless, the PL intensity was stable in a pH 3–10 aqueous solution. The as-prepared CDs applied in biomarking and bioimaging are required to be co-incubated with cells, where pH condition is around neutral (pH = 6–8), which guarantee the PL stability. Theoretically, it indicated that the prepared CDs can emit fluorescence with high stability in cells for biomarking and bioimaging.

In order to clarify the fluorescent stability of CDs, the fluorescence anti-photobleaching test was performed. As shown in Figure S2C, compared with the quantum dots (CdTe) and the traditional fluorescent dyes (DAPI), the carbon dots exhibited not only higher fluorescence intensity, but also excellent photobleaching resistance. Besides, the CDs were also well dispersed in various solutions such as DI water, PBS, FBS medium, DMEM medium, and CM1-1 medium, expecting an excellent stability in the blood system (Figure S3).

The CDs exhibited a zeta potential value of − 31.1 mV (Figure S4), which can be ascribed to the existence of oxygen and carboxyl functional groups on the surface of these particles. The potential positively charged DOX can be physically attached on the surface of CDs via electrostatic interaction with the carboxyl group and hydrophobic interaction. DOX-CDs display a zeta potential value of − 9.7 mV, confirming the fabrication of the DOX-CD complexes. Furthermore, the CDs contain an sp^2^-carbon network that can load aromatic structure of DOX via strong *π*-*π* interactions. The optimum encapsulation efficiency and drug loading efficiency were investigated by various concentrations of DOX. As demonstrated in Figure S5, the maximum encapsulation efficiency was calculated as 50.82% with the corresponding loading efficiency of 6.82% at 0.1 mg/mL of DOX.

### In Vitro Drug Release of DOX-CDs

**Fig. 2:**
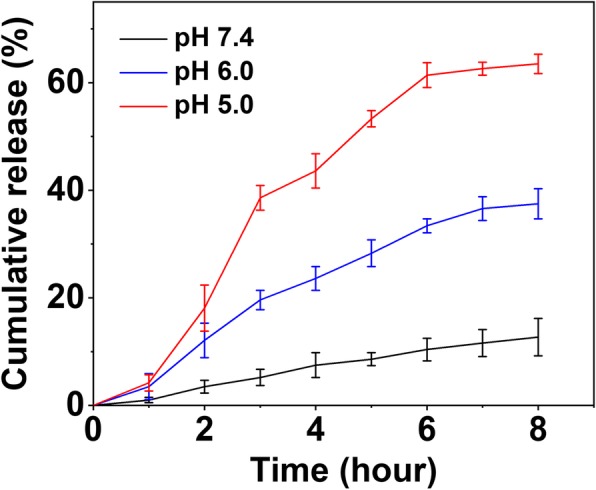
In vitro DOX release profile of DOX-CDs at pH 5.0, 6.0, and 7.4

The in vitro DOX release behavior from DOX-CDs was carried out in PBS to investigate the pH-sensitive DOX release. To demonstrate this, the DOX-CDs were incubated at different pH values (pH 7.4, 6.0, and 5.0) and the release of DOX was monitored. As shown in Fig. 2, the DOX-CDs indicated sustained release profiles in pH 7.4, 6.0, and 5.0 during the rest period. The results indicated that the DOX release was pH dependent. Only 13% of DOX was released within 8 h when the DOX-CDs were incubated at pH 7.4. However, when the pH value was lowered to 6.0 or 5.0, more than 35% or 65% of DOX was released from the DOX-CDs, respectively, suggesting the sensitivity of DOX-CDs to the low pH. It demonstrated that the amount of released DOX increased at a lower pH, which was attributed to increased protonation of –NH_2_ groups on DOX in an acidic environment. Therefore, the DOX-CDs may prohibit the premature leakage of DOX during the blood circulation and enhance the intracellular drug release. It is of great benefit to the effective treatment of cancer.

### In Vitro Cytotoxicity Test

**Fig. 3:**
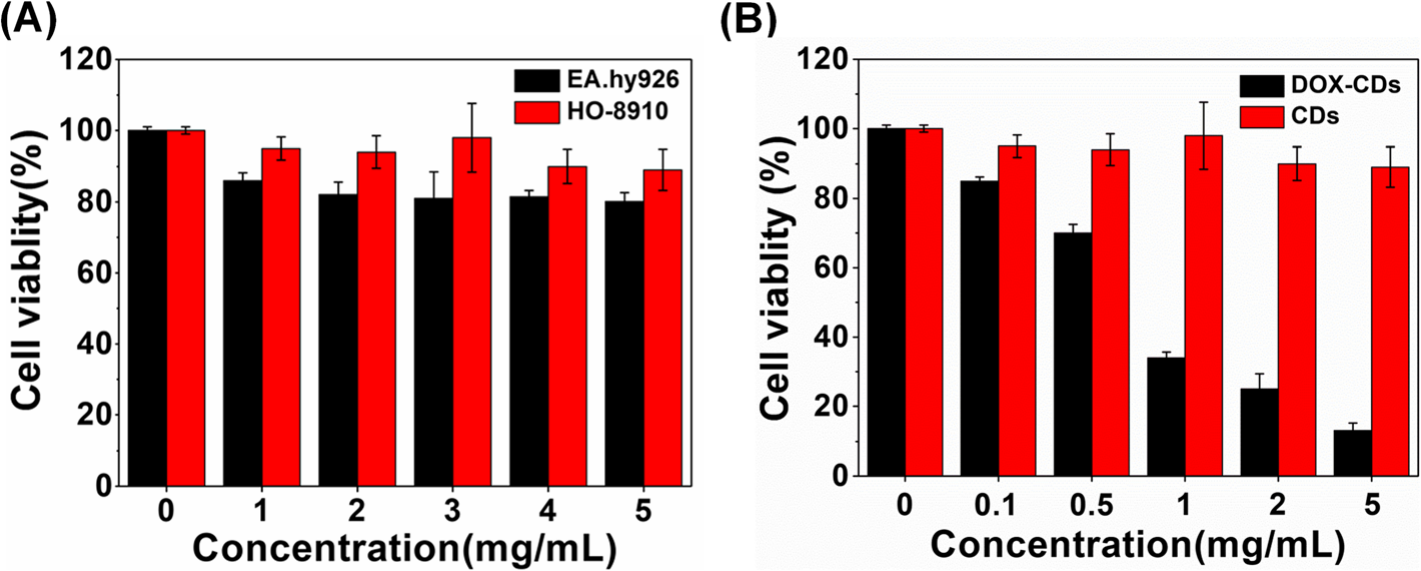
In vitro cell cytotoxicity. **a** Biocompatibility of CDs against HO-8910 and EA.hy926 cells. **b** Cell cytotoxicity of DOX-CDs and CDs against HO-8910 tumor cells. Values are expressed as mean ± SD, (*n* = 3)

The biocompatibility issue is critical for the CDs for the application in biomedical imaging and drug delivery. The cytotoxicity of CDs at various concentrations was carried out against HO-8910 ovarian cancer cells and EA.hy926 umbilical vein endothelial cells. As depicted in Fig. 3a, both the HO-8910 and EA.hy926 cells maintained high viability above 85% even at a high concentration of 5 mg/mL, indicating the excellent biocompatibility and low cytotoxicity of CDs.

When coupled with DOX, the DOX-CDs exhibited DOX concentration-dependent cell viability against the HO-8910 ovarian cancer cells. As shown in Fig. 3b, the cell viability of the DOX-CDs was significantly lower than that of DOX free CDs, especially when the concentration of DOX was above 0.05 mg/mL, indicating that excellent anticancer effect of DOX-CDs.

### In Vitro Cellular Uptake and Labeling Study

**Fig. 4:**
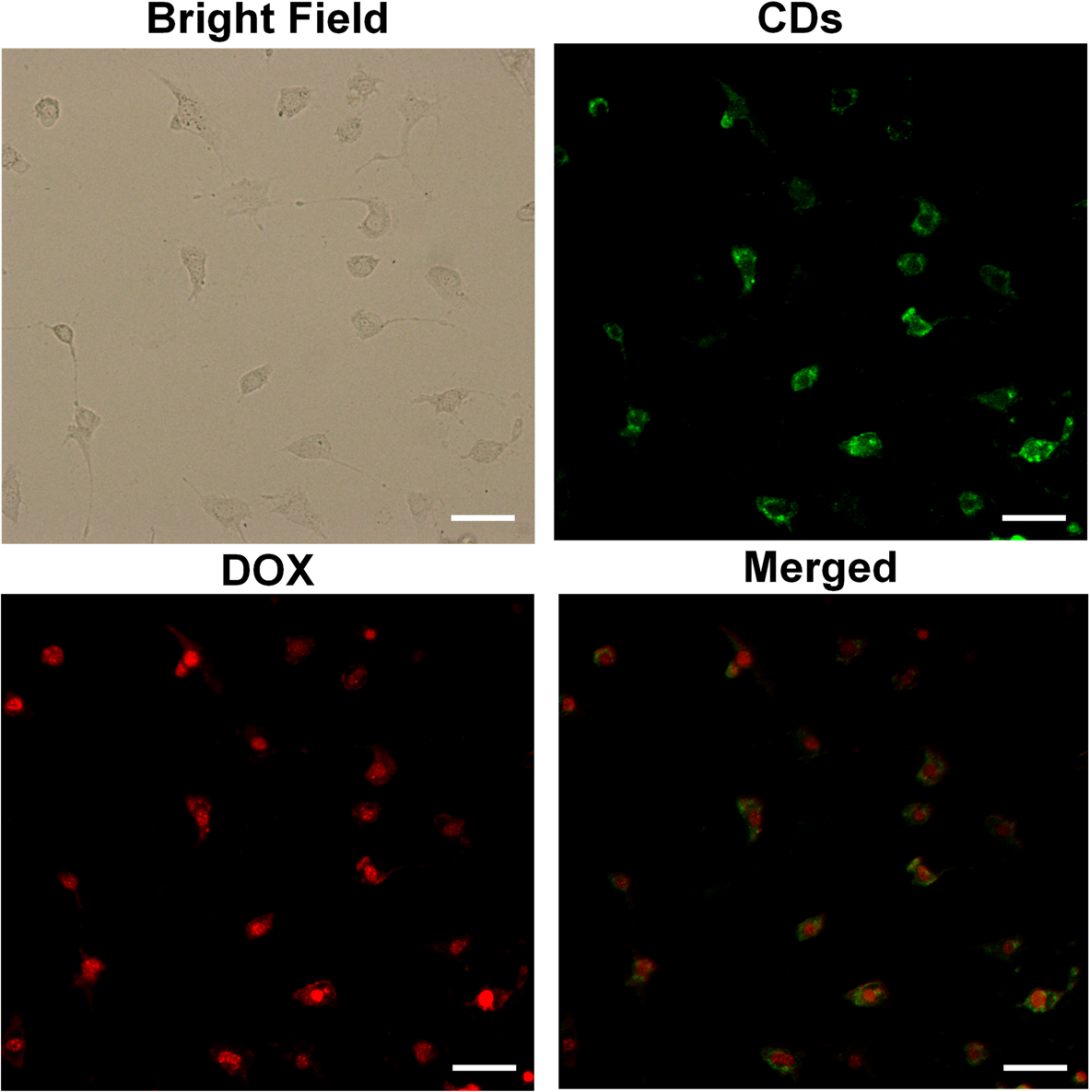
In vitro cellular uptake of DOX-CDs by HO-8910 cells. Scale bar = 50 μm

To evaluate the intracellular uptake ability of DOX-CDs, the cellular imaging was investigated on the HO-8910 cells. As illustrated in Fig. 4, vivid green and red fluorescence was observed within the cancer cells attributed to the presence of CDs and DOX, respectively. Especially, intensive green fluorescence signal mainly localized in the cytoplasm, indicating the CDs intracellular distribution. Conversely, the red signal was significantly stronger in the cell nuclei compared with cytoplasm, suggesting that the DOX may disconjugate with the CDs and moved directly to the cell nuclei attributed to its high affinity with the DNA. It could be explained that the low pH value (5.0) in the endosome and lysosome could assist the DOX release from the DOX-CDs. Therefore, the DOX-CDs could be a promising agent for cell labeling and intracellular drug delivery.

### In Vivo Animal Imaging Study

**Fig. 5:**
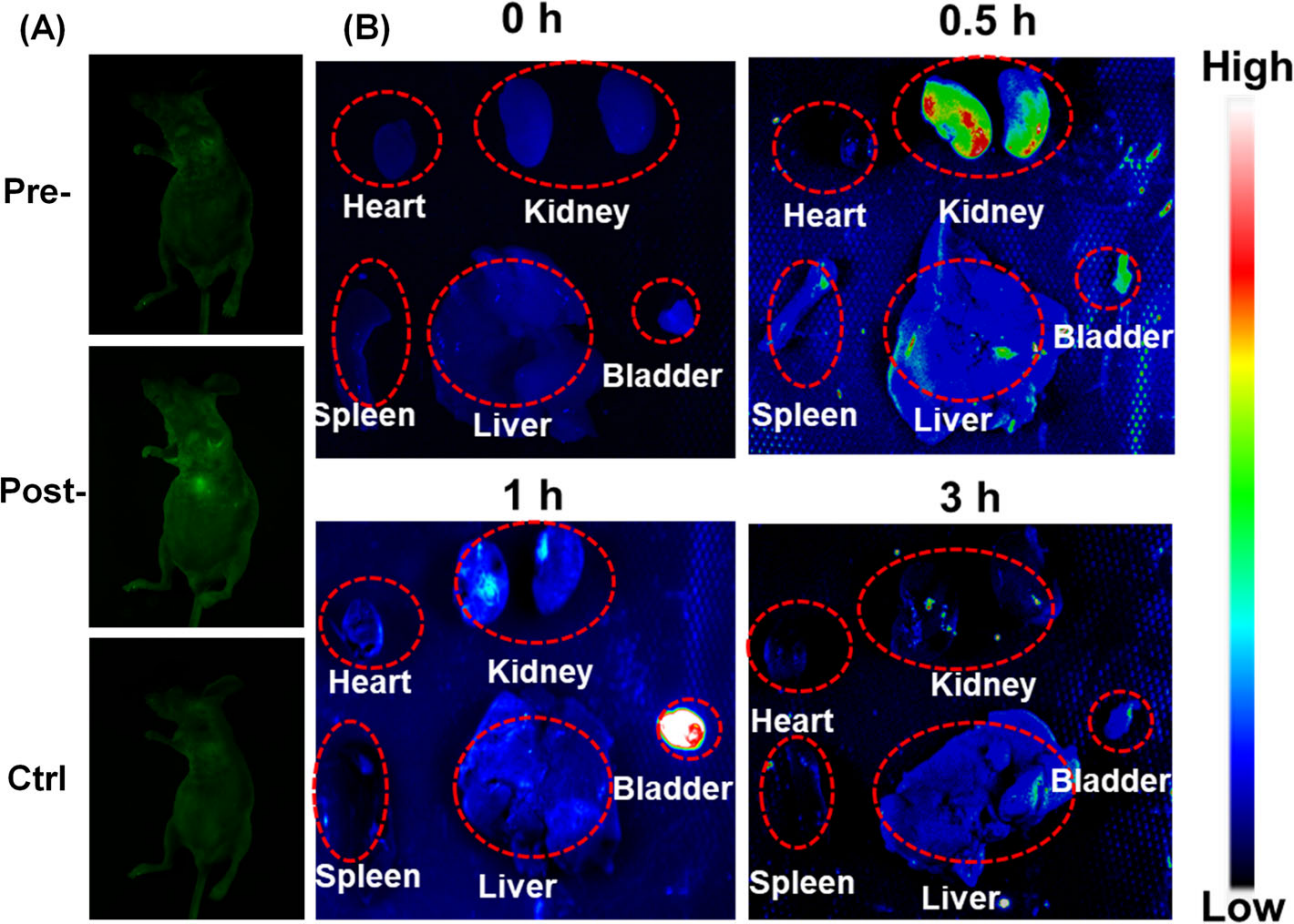
In vivo animal test. **a** Animal fluorescent imaging with CDs. **b** Ex vivo imaging of the mice after intravenous injection of CDs at different time periods

A nude mouse was subcutaneously administrated with CDs aqueous solution (50 μL, 6 mg/mL). The mouse was then anesthetized by intrapertoneal injection of 1% pentobarbital and imaged by a Tanon-5200Multi Gel imaging system under 488 nm excitation light and a 535 nm emission filter. As shown in Fig. 5a, strong green fluorescence was observed at the administrated spot, implying that the CDs fluorescence could effectively penetrate skin and tissue of mice. Moreover, the mouse maintained healthy after injections, indicating that the CDs were of excellent biocompatibility and low toxicity for animals. Considering all the results, the CDs were capable as outstanding luminescence probe for bioimaging in vitro and in vivo.

Furthermore, the biodistribution and excretion pathway of the CDs were carried out by injecting the nanoprobe via tail vein. At different time points (0, 0.5, 1, 3 h), various organs were dissected for the fluorescence imaging. As shown in Fig. 5b, the kidney and bladder revealed much stronger fluorescence signal compared with other organs including heart, spleen, and liver after injection. In addition, the fluorescence signal in kidney significantly increased within 0.5 h post-injection and gradually decreased after 1 h. Then, the fluorescence signal in bladder gradually increased from 0.5 h to 1 h, indicating that the CDs were delivered to the bladder from kidney. The result indicated that the CDs could be excreted and cleared by the kidney and bladder system.

### In Vivo Long-Term Toxicity Test

**Fig. 6:**
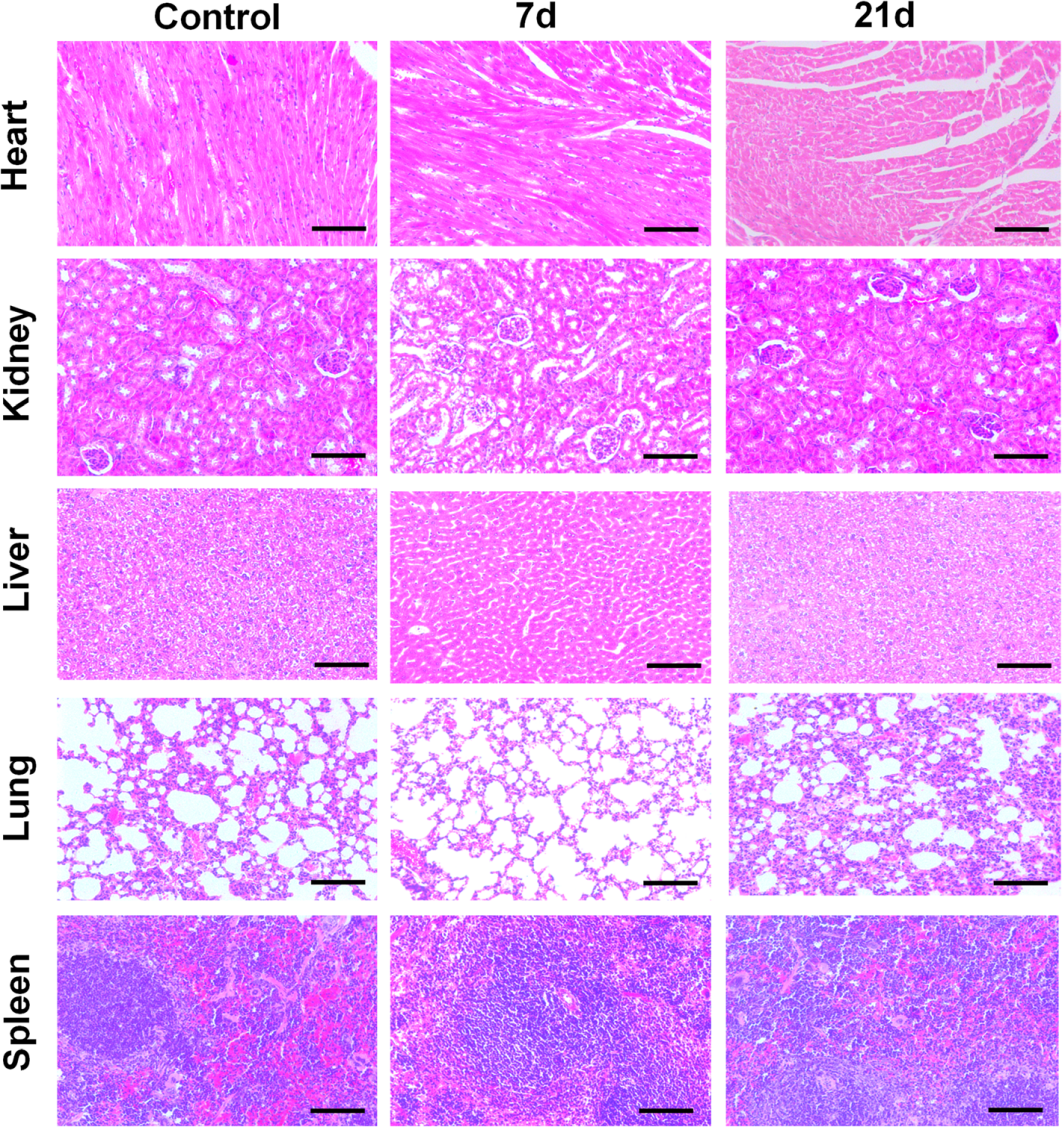
Histological analysis of major organs after 7 and 21 days administration of CDs. Scale bar = 100 μm

Furthermore, in vivo long-term toxicity study was also carried out to fully investigate the potential of employing CDs in clinical research. The Kunming mice were injected via the tail vein with PBS and CDs, and major organs (heart, lung, kidney, liver, spleen) were collected after 7 and 21 days for histological analysis. Subsequently, figures of histological tissues were imaged by a microscope to evaluate pathological difference between the experimental groups and the control group. As exhibited in Fig. 6, no remarkable organ damage and inflammatory lesion were observed in the major organs of CDs administrated animal, suggesting that the as-prepared CDs were safe for clinical use and in vivo study. Hence, the as-synthesized green CDs were biocompatible as biomarker and bioimaging probe.

## Conclusion

In conclusion, this work has demonstrated a cost-effective preparation of green fluorescent CDs with high *QY* of 93% for bioimaging and enhanced intracellular drug delivery. DOX was successfully conjugated on the CDs to form the DOX-CDs with good crystal structure, remarkable aqueous stability, and excellent photoluminescence property. The DOX-CDs could respond to the intracellular pH environments to promote acid-triggered intracellular release. Attributed to the pH sensitivity, the DOX-CDs showed effective inhibition to the proliferation of HO-8910 cells. The DOX-CDs exhibited excellent cell labeling ability and responded to the endo-/lysosomal pH to release the DOX within the cells. The CDs acted as fluorescent probes both in vitro and in vivo. Finally, no remarkable toxic effect was observed from the CD-treated mice by the histological analysis. Nevertheless, the work demonstrated that CDs prepared by the cost-effective method may have great potential in biomedical imaging and intracellular drug delivery.

## Supplementary information

**Additional file 1: Figure S1.** XPS spectrum of DOX-CDs: (A) C1s spectrum, (B) O1s spectrum; **Figure S2.** Stability test of DOX-CDs at (A) Different time, (B) different pH, (C) Fluorescence anti-photobleaching test; **Figure S3.** Bright and fluorescent photos of DOX-CDs in various medium including DI water, PBS, FBS, DMEM and CM1-1; **Figure S4.** The zeta potential of CDs, DOX and DOX-CDs; **Figure S5.** Drug loading ability of CDs: (A) Drug encapsulation efficiency at various concentrations of DOX, (B) Drug loading efficiency at various concentrations of DOX

## Data Availability

All data generated or analyzed during this study are included in this published article and its supplementary information files.

## Abbreviations

CDs: Carbon dots
DOX: Doxorubicin
DOX-CDs: Doxorubicin entrapped carbon dots
FBS: Fetal bovine serum
MTS assay: MTS Cell Proliferation Colorimetric Assay Kit
PBS: Phosphate-buffered saline
*QY*: Quantum yield
TEM: Transmission electron microscopy
XPS: X-ray photoelectron spectroscopy
