# Supplementary material for: The Cost-Effective Preparation of Green Fluorescent Carbon Dots for Bioimaging and Enhanced Intracellular Drug Delivery
Source: Nanoscale Res Lett. 2020 Mar 4;15:55. doi: 10.1186/s11671-020-3288-0 (PMC7056761; doi:10.1186/s11671-020-3288-0)
Supplement: Supplementary file 1 — Additional file 1: Figure S1. XPS spectrum of DOX-CDs: (A) C1s spectrum, (B) O1s spectrum; Figure S2. Stability test of DOX-CDs at (A) Different time, (B) different pH, (C) Fluorescence anti-photobleaching test; Figure S3. Bright and fluorescent photos of DOX-CDs in various medium including DI water, PBS, FBS, DMEM and CM1-1; Figure S4. The zeta potential of CDs, DOX and DOX-CDs; Figure S5. Drug loading ability of CDs: (A) Drug encapsulation efficiency at various concentrations of DOX, (B) Drug loading efficiency at various concentrations of DOX [file 11671_2020_3288_MOESM1_ESM.docx]

Supplementary Data

**The cost-effective preparation of green fluorescent carbon dots for bioimaging and enhanced intracellular drug delivery**

Yuqing Sun^a,1^, Shaohui Zheng^a,b,1*^, Long Liu^a^, Ying Kong^a^, Aiwei Zhang^b^, Kai Xu^a,b*^, Cuiping Han^a,b*^

^a^School of Medical Imaging, Xuzhou Medical University, Xuzhou, Jiangsu,221004, PR China

^b^Department of Radiology, Affiliated Hospital of Xuzhou Medical University, Xuzhou, Jiangsu221000, PR China

^*^Corresponding authors: C. Han (hancp@xzhmu.edu.cn) and K. Xu ([xkpaper@163.com](mailto:xkpaper@163.com)) S. Zheng (shaohui19910@163.com)


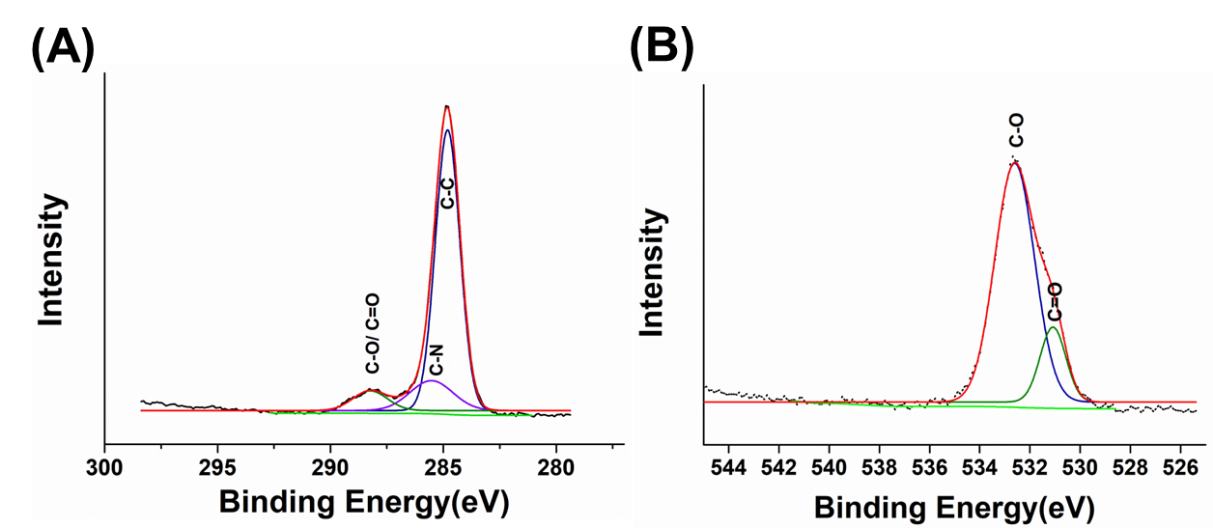


**Figure. S1** XPS spectrum of DOX-CDs: (A) C_1s_ spectrum, (B) O_1s_ spectrum


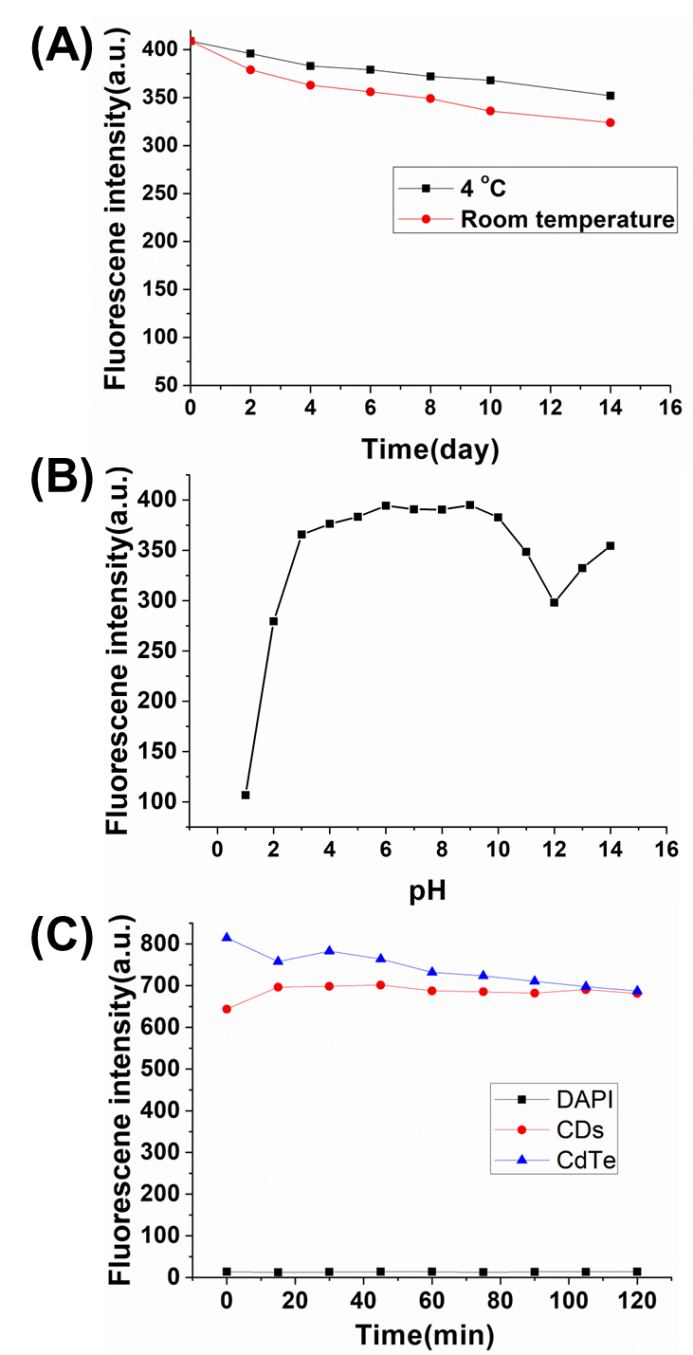


**Figure. S2** Stability test of DOX-CDs at (A) Different time, (B) different pH, (C) Fluorescence anti-photobleaching test.

**
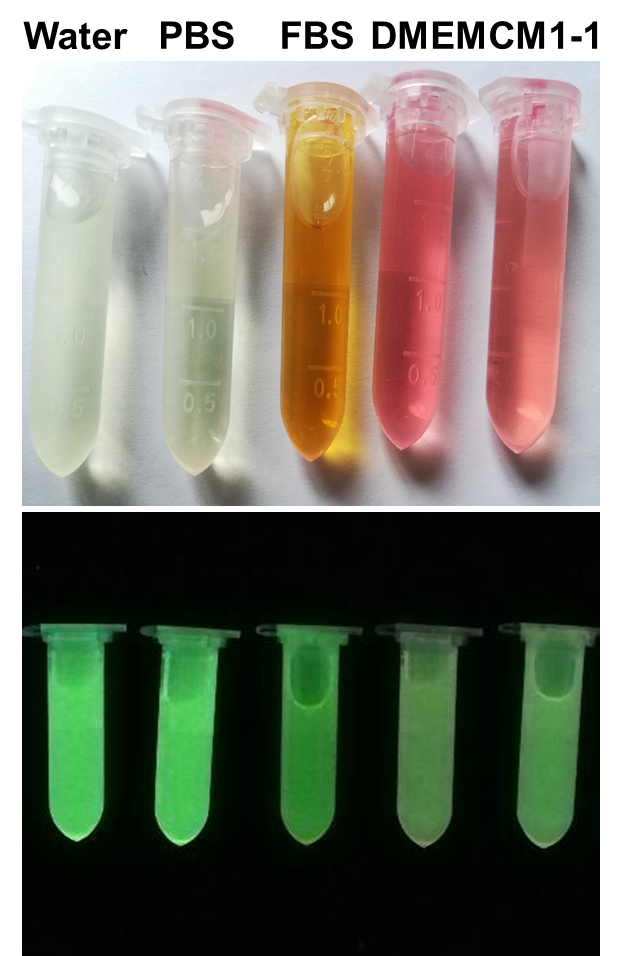
**

**Figure. S3** Bright and fluorescent photos of DOX-CDs in various medium including DI water, PBS, FBS, DMEM and CM1-1.

Figure. S4. The zeta potential of CDs, DOX and DOX-CDs.

**Figure. S5** Drug loading ability of CDs: (A) Drug encapsulation efficiency at various concentrations of DOX, (B) Drug loading efficiency at various concentrations of DOX.
